# Breast Lesions of Uncertain Malignant Potential and Risk of Breast Cancer Development: A Single-Center Experience on 10,531 Consecutive Biopsies

**DOI:** 10.3390/medicina61101877

**Published:** 2025-10-20

**Authors:** Maria Orsaria, Alessandro Mangogna, Massimo Bertoli, Carla Di Loreto, Enrico Pegolo

**Affiliations:** 1 Institute of Pathology, Azienda Sanitaria Universitaria Friuli Centrale, University Hospital of Udine, 33100 Udine, Italy; maria.orsaria@asufc.sanita.fvg.it (M.O.); carla.diloreto@uniud.it (C.D.L.); enrico.pegolo@asufc.sanita.fvg.it (E.P.); 2 Department of Medicine (DMED), University of Udine, 33100 Udine, Italy

**Keywords:** B3 lesions, malignant potential, surgical excision, upgrade rate, follow-up

## Abstract

*Background and Objectives*: Breast lesions of uncertain malignant potential identified on biopsy, known as “B3 lesions,” constitute a significant portion of diagnoses in numerous published studies. These lesions are associated with a variable risk of coinciding malignant tumors, and current guidelines recommend complete excision, which can occasionally lead to an upgrade in the resection specimen. However, alternative, less invasive treatment strategies, such as clinical follow-up, may be considered. In this study, we retrospectively analyzed diagnostic biopsies from our institution to determine the upgrade rate of each B3 lesion subgroup to breast malignancy following complete excision. *Materials and Methods*: All breast biopsies conducted at our institution from 1 January 2018 to 30 November 2022 and classified as B3 lesions were included in this study. The lesions were categorized into groups and subgroups based on their growth pattern and histopathological features. To determine the upgrade rate to ductal carcinoma in situ (DCIS) or invasive breast cancer (IBC) for each B3 lesion subgroup, we assessed the histological concordance between the biopsy and the resection specimen. *Results*: During the study period, 10,531 biopsies were performed, of which 1045 (9.93%) were classified as B3 lesions. Among these, 795 (76.08%) were subsequently resected, either through surgical procedures (98.32%) or using the Vacuum-Assisted Excision technique (1.68%). Histological examination revealed that 89 (11.19%) of the resected B3 lesions were upgraded to breast malignancy, with 59 cases (7.42%) progressing to DCIS, 22 cases (2.76%) to IBC, and 8 cases (1.01%) to borderline or malignant phyllodes tumor. The upgrade rate varied among histopathological subgroups, being lowest in complex sclerosing lesions without atypia (4.95%, 95% CI: 2.5–8.7%) and highest in intraductal papillomas with atypia (58.82%; 95% CI: 32.9–81.6%). *Conclusions*: Statistically significant differences were observed between B3 lesion subgroups, with a higher risk of upgrade in lesions exhibiting atypia. As our understanding of B3 lesions evolves, there is potential to implement therapeutic strategies tailored to the specific risk associated with each subgroup. This approach could allow for less invasive management options, such as clinical or radiological follow-up, thereby sparing patients from unnecessary invasive procedures when appropriate.

## 1. Introduction

Breast cancer continues to be the most diagnosed neoplasm in women [1,2]. The high incidence of this tumor has drastically attracted research interest over the years and led to a progressive improvement of diagnostic techniques, with particular attention to screening and enhanced conservative surgery. However, improved screening and subsequent radiological and surgical interventions have caused an inevitable “over-diagnosis” risk: even though not all early-diagnosed lesions will then evolve into invasive neoplasms, their identification in early stages may determine the beginning of a clinical pathway like that of patients with confirmed invasive tumors. An example is constituted by the ductal carcinoma in situ (DCIS), which is often diagnosed in asymptomatic patients and is treated according to current guidelines with a combination of surgery, radiotherapy, and endocrine therapy, despite only 20–30% of these lesions will progress towards invasive tumors [3]. Thus, “over-diagnosis” leads to “over-treatment” of many lesions that would never have caused any harm sufficient to require treatment.

Over-diagnosis and over-treatment are unresolved issues of particular concern in lesions of uncertain malignant potential, known as “B3 lesions”, since they may lead to potentially unnecessary therapeutic interventions [4,5]. The incidence of breast biopsies classified as B3 lesions varies wildly among different institutions, countries, and studies. The range of 3% to 12%, with some studies reporting up to 21%, highlights the variability in the prevalence of these lesions across different populations and healthcare settings [6,7,8,9,10,11,12,13,14,15]. These lesions are well-characterized from a histological point of view but display features of heterogeneous presentation, including very different entities, with different progression potential: in fact, they may show benign characteristics in the portion sampled which is not representative of the entire lesion, or they may be associated with malignant lesions such as DCIS or invasive carcinoma [16,17,18]. Therefore, the risk is not only represented by a possible evolution of the lesion but rather by a simultaneous presence of lesions of malignant nature. Diagnosis and management are also complicated by the heterogeneity of B3 lesions, comprising lesions with or without histopathological atypia: intraductal papillary lesion (intraductal PL), radial scar/complex sclerosing lesion (RS-CSL), phyllodes tumor (PT), flat epithelial atypia (FEA), atypical ductal hyperplasia (ADH), atypical lobular hyperplasia (ALH), lobular carcinoma in situ (LCIS), and other miscellaneous entities, including mucocele-like lesions, adenomyoepitheliomas, microglandular atypical adenosis (AMGA), and atypical apocrine adenosis (AAA) [16,17,18,19]. The management of B3 lesions has undergone significant changes, remaining a noteworthy challenge in clinical practice. The traditional approach is represented by open surgical excision due to the uncertain risk of malignancy and concerns about the adequacy of image-guided sampling techniques [8]. The rationale for this procedure is to identify any coincident significant malignant disease not detected by the previous biopsy [11,20,21]. However, it is increasingly recognized that some B3 subtypes are associated with a negligible risk of malignancy, and the rate of progression occurs over a long time [22,23]. Therefore, open surgical excision for all, without any distinction, would constitute an over-treatment, mainly because the majority of B3 lesions at excision lead back to benignity [24].

The current lack of standardized management and the existence of different guidelines developed by breast societies underscores the need for further research in this area to guide clinical practice.

The aim of this study was to characterize the histopathological features, distributions, and upgrade rates of B3 lesion subtypes, thus suggesting the best approach for each type of lesion.

## 2. Materials and Methods

This retrospective, observational, single-center study was approved by the Institutional Review Board of the Department of Medicine—University of Udine (approval number 037/2019_IRB) on 9 September 2019. Due to the retrospective nature of the study, obtaining informed consent from patients was not required. All consecutive histology reports of breast biopsies signed out from 1 January 2018, until 30 November 2022, were retrieved from the Laboratory Information System software (INSIEL APSys version 9.0, Insiel S.p.A., Trieste, Italy) of the Institute of Pathology of the University Hospital of Udine, “Azienda Sanitaria Universitaria Friuli Centrale”. Initial sampling was performed either by ultrasound (US)-guided 14-gauge (G) core needle biopsies (CNBs), with at least three samples taken, or X-ray-guided 8–11 G needles vacuum-assisted biopsy (VAB), with a median of 12 cores collected during each procedure. All histological diagnoses performed on breast biopsies were classified according to the B-coding system, employed by the United Kingdom’s National Health Service—Breast Cancer Screening program, as follows: B1, normal tissue or non-diagnostic/unsatisfactory; B2, benign lesion; B3, lesion of uncertain malignant potential; B4, lesion suspicious for malignancy; or B5, malignant lesion [11,25].

From the initial dataset, all cases with a B3 lesion diagnosis were selected and included in this study. Furthermore, the B3 lesions were divided into main subgroups according to their histopathology and growth pattern: nodular proliferative epithelial lesions (with and without atypia), diffuse/non-nodular atypical proliferative epithelial lesions, myoepithelial tumors, fibroepithelial neoplasms (with stromal hypercellularity, with epithelial or stromal atypia) and mesenchymal tumors [16,17,18,19].

Following a B3 diagnosis, most of the lesions were resected either surgically (no-dulectomy) or, less frequently, radiologically [vacuum-assisted excision (VAE) technique]. During the study period, three pathologists participated in diagnosing all the breast specimens, including one with over 30 years of experience in breast pathology (C.D.L.) and two others with a minimum of 10 years of experience in the same field (M.O., E.P.). Most cases were signed out independently by a single pathologist, with only a very limited set of complex cases being signed out collegially.

The following data were recorded for each patient: age; follow-up period; radiological features of the lesion—categorized in nodule, microcalcifications, nodule with microcalcifications and duct ectasia; Breast Imaging Reporting & Data System (BI-RADS^®^) category; type of biopsy distinguished in CNB or VAB; histological diagnosis in the biopsy material and in the corresponding resection specimen.

Upgrade rates were calculated for each B3 lesion when the resection specimen was available. Ninety-seven B3 lesions, diagnosed in patients with concomitant breast cancer during the workup for the assessment of the extent of the disease and subsequently excised, were excluded from the study because surgery would have been performed nonetheless, dictated by the diagnosis of malignancy, therefore introducing a clinical bias. Confidence intervals (CIs) were calculated to establish the reliability of the upgrade rates. We conducted additional analysis to assess if there were statistically significant differences in the upgrade rates among different types and subgroups of B3 lesions using Fisher’s exact test. The statistical significance was set to a *p*-value < 0.05. Statistical analyses were performed using GraphPad Prism version 10.3.1 for Mac OS X, GraphPad Software, Boston, MA, USA.

## 3. Results

In the study period, a total of 10,531 breast biopsies were performed, 1045 (9.93%) of which were classified in the B3 category. The distribution of lesions using the B-coding system is shown in Table 1. The mean age of the patients with a B3 diagnosis was 55.15 years, with a standard deviation of 14.61 (range from 17 to 98 years). Patients’ follow-up period ranged from 1 to 59 months (mean, 29.1; median, 30).

The radiological appearance of the lesions was 652 nodules, 34 nodules with microcalcifications, 189 were microcalcifications only, and 25 were ductal ectasias; there was no radiological information for the remaining 145 cases (Table 2).

Most B3 lesions were detected with an ultrasound-assisted high-speed CNB (804 cases; 76.94%), and 241 B3 lesions (23.06%) were detected with stereotactic VAB (Table 2). A total of 892 B3 lesions were resected (85.36%); of these, the majority surgically (98.32%), less frequently with the VAE technique (1.68%) (Table 2). Ninety-seven of the resected lesions were identified during workup for an already diagnosed breast malignancy and were excluded from the study.

Among B3 lesions, 243 cases (23.25%) were non-epithelial proliferative lesions, as shown in Table 3; fibroadenoma with stromal hypercellularity was lesions more frequent (176 cases; 72.43%).

Most epithelial proliferative lesions had a nodular radiological appearance (606 cases; 57.99%), whereas a diffuse/non-nodular aspect was only in 196 cases (18.75%); atypia was also present in the latter cases. Among nodular proliferative epithelial lesions, no atypia was detected in 537 cases (88.6%), while atypia was identified in 69 cases (11.39%) (Table 3).

An overview of the histological features of all proliferative epithelial B3 lesions (802 cases; 76.75%) is reported in Table 4.

Nodular proliferative epithelial lesions were diagnosed as intraductal PLs and RS-CSLs, subsequently divided into lesions without/with atypia. RS-CSL was the most frequent lesion with 329 cases (31.5%), with nodular radiological appearance, of which the majority lacked atypia (286 cases; 86.9% of all RS-CSLs), while only a part showed atypia on histological examination (43 cases; 13.1% of all RS-CSLs). Between intraductal PLs, characterized by branches of proliferative fibrovascular tissue topped with a layer of epithelial and myoepithelial cells, only 26 cases (9.4% of all intraductal PLs) presented atypia. One hundred and ninety-six biopsies (18.75%) were diagnosed as diffuse/non-nodular atypical proliferative epithelial lesions containing mostly ADH in 92 cases (47%) and ALH in 65 cases (33.2%). FEA, identified histopathologically by the atypical proliferation of epithelial cells without architectural abnormalities, and classical LCIS, consisting of various atypical epithelial proliferation originating from the acinar structures of the breast, were observed in 16 biopsies (8.2%) and 21 biopsies (10.6%), respectively (Table 4).

**Figure 1:**
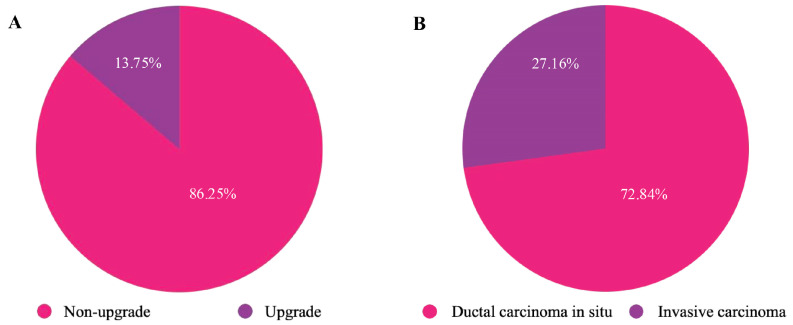
(**A**) Percentage of non-upgrade and upgrade for the whole group of epithelial proliferative B3 lesions after resection. (**B**) Global percentage of upgrade to non-invasive and invasive carcinomas.

Out of 802 cases of epithelial proliferative B3 lesions, a total of 589 cases (73.44%) underwent surgical resection, 403 cases (68.4%) of nodular proliferative epithelial lesions (without atypia), 50 cases (8.5%) of nodular proliferative epithelial lesions (with atypia), and 136 cases (23.1%) of diffuse/non-nodular atypical proliferative epithelial lesions, respectively. In total, 81 epithelial proliferative B3 lesions (13.58%) were upgraded to a malignant lesion (Table 5, Figure 1A), and among these there were 22 cases (27.50%) of invasive carcinomas (Table 5, Figure 1B). About the 243 non-epithelial B3 lesions, 206 cases (84.77%) underwent surgical resection, with 8 cases (3.88%) upgraded to malignant lesions, mainly borderline or malignant phyllodes tumors (Table 6).

**Figure 2:**
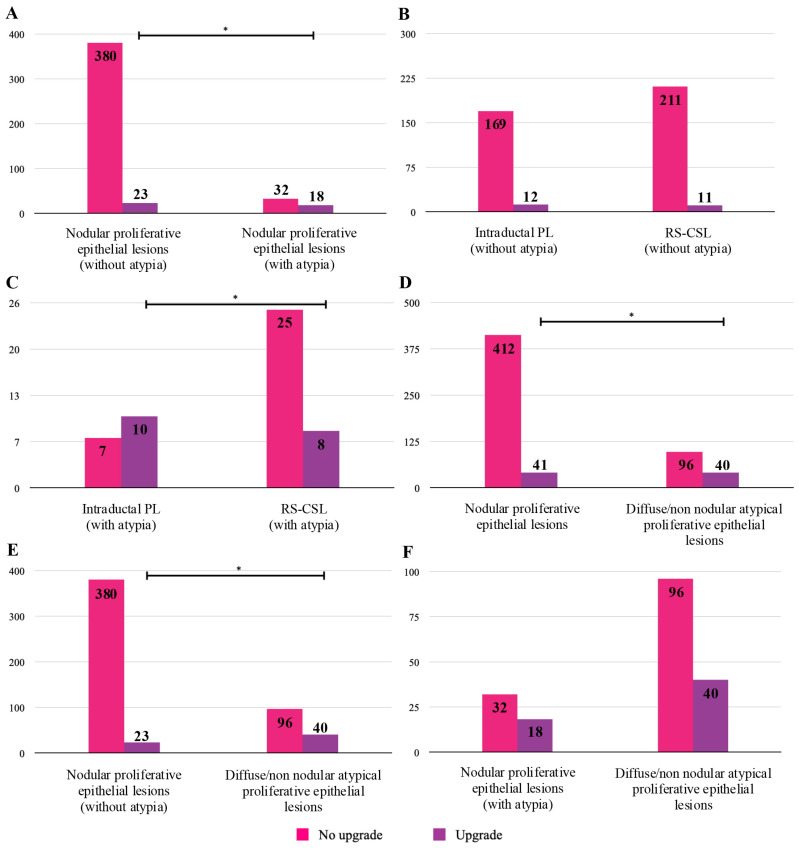
Statistical difference between the upgrade rates of epithelial proliferative B3 lesions (*: *p*-value < 0.05).

Figure 2 shows the differences between the upgrade rates of epithelial proliferative B3 lesions. A significant correlation was found between the detection of atypia in a B3 lesion biopsy and an upgrade to breast malignancy in the resection specimen, regardless of the radiological appearance of the lesion (nodular or diffuse/non-nodular proliferative epithelial lesions) (*p* < 0.05) (Figure 2A,D,E). Among the nodular proliferative epithelial B3 lesions, a significant difference in upgrade rates was found when considering intraductal PL vs. RS-CSL, always in the presence of atypia (Figure 2C).

Each subgroup of diffuse/non-nodular atypical proliferative epithelial B3 lesions was compared individually with the other subgroups to see if there was a significantly different upgrade rate to each group. Fisher’s exact test showed that the upgrade rate of ADH was significantly different to that of ALH (*p* = 0.0206). No further significant differences have been found between other subgroups (Table 7).

Lastly, invasive carcinomas diagnosed in excision specimens were mainly low-grade and had a luminal phenotype (Table 8).

## 4. Discussion

The B-coding system, employed by the National Health Service—Breast Cancer Screening program, classifies breast biopsy results into five diagnostic categories: B1, normal tissue or non-diagnostic/unsatisfactory; B2, benign lesion; B3, lesion of uncertain malignant potential; B4, lesion suspicious for malignancy; and B5, malignant lesion [11,25]. Within this framework, our large retrospective study analyzing 1045 B3 lesions out of 10,531 consecutive breast biopsies offers valuable real-world insights into the biological behavior and malignancy potential of these diagnostically challenging lesions. Compared with previous multicenter studies—such as the Italian VAB consortium report by Bianchi et al. [8], which included 584 B3 lesions collected across multiple institutions, and the Swiss registry by Saladin et al. [9], based on pooled vacuum-assisted biopsies—our work offers the advantage of a completely homogeneous diagnostic workflow. All 1045 B3 lesions in our cohort were evaluated within a single pathology department, using uniform sampling techniques and reporting criteria according to the NHS B-coding system. This minimizes inter-institutional variability and allows a more reliable comparison of upgrade risk across different histopathological subtypes.

The incidence of B3 lesions in our cohort (9.93%) is in line with previous reports, which describe a varying prevalence, ranging from 3% to 12%, and occasionally up to 21%, reflecting institutional, geographical, and population-specific differences [6,7,8,9,10,11,12,13,14,15]. The overall upgrade rate to malignancy in our series (11.19%) also falls within the lower end of the reported range of 10–30% [7,8,11,13,14,21]. Crucially, our results underscore the pivotal role of histopathological subtyping—particularly the presence or absence of cytological atypia—in risk stratification. When pooled by cytological atypia, B3 lesions with atypia showed a markedly higher upgrade rate compared to those without atypia (36.0% vs. 5.7%), as detailed in Table 5. Among lesion subtypes, intraductal PLs with atypia demonstrated the highest upgrade rate (58.8%), confirming previous findings that associate atypia with increased malignancy risk [7,11,14,20]. Conversely, RS-CSLs without atypia exhibited a significantly lower risk of upgrade (4.95%), supporting a more conservative approach for these lesions.

In addition, our data show that diffuse/non-nodular atypical epithelial proliferations—especially ADH and LCIS—presented considerable upgrade rates (38.5% and 16.7%, respectively), reinforcing their classification as higher-risk entities, as previously suggested in the literature [4,5,20,23]. In contrast, FEA and ALH displayed more variable behaviors, with upgrade rates that did not differ significantly from each other [21]. It should be noted that some histopathological subgroups in our series—such as atypical microglandular adenosis (AMGA) and atypical apocrine adenosis (AAA)—were represented by very few cases. Accordingly, their upgrade rates cannot be considered statistically reliable and are reported only for descriptive completeness rather than to support standalone conclusions.

A further point of interest emerges from the statistical comparisons among diffuse/non-nodular atypical epithelial B3 subtypes, as detailed in Table 7. Among all comparisons, only the difference in upgrade rates between ADH and ALH reached statistical significance (*p* = 0.0206). This finding aligns with the more clearly established relationship between ADH and malignant progression [4,20], and it emphasizes ADH as a lesion that may require surgical excision. The lack of significant differences between other subtypes, such as FEA and LCIS, could be attributed to limited sample sizes or overlapping biological behaviors. Altogether, these results highlight the importance of precise histological interpretation within the B3 spectrum and suggest that ADH, in particular, should remain classified as a high-risk lesion, warranting surgical excision or close radiological follow-up.

Importantly, our analysis also revealed that nearly all carcinomas identified at definitive histology in B3 lesions were low-grade and exhibited a luminal immunophenotype, indicating a generally favorable biological profile [22,23]. Specifically, most invasive carcinomas were low grade (G1–G2), luminal A subtype, and predominantly pT1 suggesting, from a clinical point of view, that a substantial proportion of these lesions may be adequately managed with breast-conserving surgery followed by endocrine therapy alone, without the need for chemotherapy or extensive axillary intervention. This reinforces the concept that, although upgrade events do occur, their biological behaviour is often indolent, supporting risk-adapted rather than universally aggressive management.

Another notable finding relates to the diagnostic concordance between the initial biopsy and the subsequent resection specimen, as shown in Table 5. In certain subgroups, especially nodular proliferative lesions without atypia, concordance rates were lower than anticipated. Indeed, in a proportion of these resections no residual lesion was identified, possibly due to complete removal at the time of the initial biopsy. This scenario has been described previously in literature and is thought to reflect the growing precision of image-guided biopsy techniques [11,14]. While this may lower apparent concordance rates, it also lends support to less invasive strategies, provided that pre-operative sampling is adequate and concordant with radiological findings.

The importance of personalized management strategies for B3 lesions in order to avoid unnecessary overtreatment has been emphasized in recent international guidelines. The 2023 EUSOMA consensus guidelines and the 3rd International Consensus Conference on B3 lesions advocate for more conservative alternatives—such as VAE or imaging surveillance—in selected cases, particularly in lesions without atypia and with radiologically benign features [15,18]. Our data strongly support this direction: for example, nodular epithelial lesions without atypia (such as benign PLs or RS-CSLs) showed low upgrade risks and may be appropriately managed with VAE, potentially sparing patients from unnecessary surgical excision [13,15,24].

In addition to these recent European recommendations, our diagnostic workflow and clinical decision-making were also informed by the 2018 NHS Breast Screening multidisciplinary guidelines [11], which remain one of the most comprehensive and widely used references for B3 lesion classification and management. The work of Pinder et al. provides critical guidance in determining which B3 subtypes require excision versus those suitable for conservative follow-up, and this framework was instrumental in shaping our institution’s approach [11].

Our study also addressed fibroepithelial B3 lesions, a category that is less extensively discussed in the literature. In our cohort, 8 of these lesions were upgraded to borderline or frankly malignant phyllodes tumor, while 18 were reclassified at resection as benign phyllodes tumor, an entity that is unlikely to progress to aggressive malignancy and, as such, may have limited prognostic impact and may be managed conservatively in selected patients [16,19].

From a procedural standpoint, ultrasound-guided CNB was the primary diagnostic method in our series (77%), followed by stereotactic VAB. Although a direct comparison of upgrade rates between techniques was beyond the scope of our study, the literature suggests that VAB may offer more representative tissue sampling, particularly in the context of microcalcification-dominant lesions, and could therefore reduce the risk of underdiagnosis [8,9,12,14].

In our cohort, radiological appearance of the lesion (nodule, microcalcifications, nodule with microcalcifications or duct ectasia) did not demonstrate a significant association with upgrade risk. This suggests that radiological patterns per se may have limited predictive value unless integrated with histopathological information. Prospective studies analysing radiological-pathological correlation are needed to establish the role of imaging features in risk stratification of B3 lesions.

A key strength of our study lies in the size and consistency of the cohort. Unlike most multicenter reports—where variability in sampling techniques, pathology reporting, and excision policies may influence upgrade estimates—our 1045 B3 lesions were collected consecutively and managed within a single institution. This homogeneous framework allowed us to compare upgrade risks across several histopathological subtypes under strictly uniform diagnostic conditions, providing one of the most internally consistent real-world datasets available to date. We believe this contributes meaningful confirmation to current guideline recommendations, particularly regarding the differential impact of atypia on upgrade risk.

This study has several limitations. Its retrospective and single-center design may limit generalizability, and a potential referral bias cannot be excluded, as a proportion of patients may have continued management at external oncology institutions after the diagnostic biopsy (153 out of the 1045 B3 biopsied B3 lesions did not undergo excision in our institution). Moreover, 97 B3 lesions identified synchronously during staging for an already diagnosed breast carcinoma were intentionally excluded, as their treatment pathway was dictated by the index malignancy. However, the absence of excision data for these lesions prevents assessment of their upgrade rate and may have introduced an element of selection bias. The relatively short follow-up period of our cohort may not be sufficient to capture late malignant events, particularly in indolent B3 subtypes; longer prospective surveillance would be required to fully assess long-term outcomes and the true natural history of these lesions. These limitations should be considered when interpreting the findings, which nonetheless derive from one of the largest consecutively collected single-institution B3 cohorts currently available.

## 5. Conclusions

This large single-center study reinforces the complexity and heterogeneity of B3 breast lesions, revealing that not all carry the same risk—or require the same response. Our findings clearly demonstrate that histopathological features, particularly the presence of atypia, are central to predicting malignant upgrade. While certain lesions such as intraductal PLs with atypia demand heightened clinical attention, others—like CSLs without atypia—showed minimal malignant potential.

Crucially, most upgraded lesions were low-grade carcinomas with a favorable luminal phenotype, indicating that aggressive treatment may not always be necessary. These insights challenge the traditional “one-size-fits-all” approach and support a paradigm shift toward tailored, risk-adapted management.

We advocate for broader adoption of minimally invasive strategies—such as VAE—for carefully selected low-risk lesions. This approach not only maintains diagnostic safety but also reduces overtreatment, surgical morbidity, and patient anxiety.

While our findings support a risk-adapted approach to B3 lesions, they warrant confirmation in prospective multicenter studies. Harmonization of diagnostic and management criteria across institutions will be essential to translate these observations into standardized clinical practice. In the era of precision medicine, B3 lesions must be managed not only with caution—but with evidence, and patient-centered foresight.

## Figures and Tables

**Table 1:** Patient distribution by biopsy lesions.

Diagnosis	Nr. Patients	Percentage (%)	
B1: Non-diagnostic/unsatisfactory or normal tissue only	311	2.95	
B2: Benign lesion	5480	52.03	
B3: Lesion of uncertain malignant potential	1045	9.93	
B4: Suspicion of malignancy	48	0.45	
B5a: Malignant (non-invasive)	446	4.24	34.64
B5b: Malignant (invasive)	3103	29.47	
B5c: Malignant (in situ/invasion not assessable)	77	0.73	
B5d: Malignant (non-epithelial, metastatic)	21	0.2	
Total 10,531			

**Table 2:** Clinical and radiological features of the B3 lesions case series of this study.

	Nr. Patients	Percentage (%)
Radiological feature	900	86.1
- Nodule	652	62.39 (72.5)
- Microcalcifications	189	18.09 (21.1)
- Nodule with microcalcifications	34	3.21 (3.7)
- Duct ectasia	25	2.39 (2.7)
Radiological feature not indicated	145	13.9
BI-RADS category	956	91.5
- R2	1	0.1 (0.1)
- R3	266	25.25 (27.8)
- R4	673	64.40 (70.43)
- R5	16	1.53 (1.67)
BI-RADS category not indicated	89	8.5
Type of biopsy		
- Core needle biopsy	804	76.94
- VAB	241	23.06
Type of excision	892	85.36
- Open excision	877	83.92 (98.32)
- VAE	15	1.44 (1.68)
No excision	153	14.64

Abbreviation: BI-RADS, Breast Imaging Report and Data System; R, radiography; VAB, vacuum assisted biopsy; VAE, vacuum-assisted excision.

**Table 3:** Histological features of the B3 lesions included in the study.

	Nr. Lesions	Percentage (%)
Nodular proliferative epithelial lesions	606	57.99
- without atypia	537	51.4 (88.61)
- with atypia	69	6.6 (11.39)
Diffuse/non-nodular atypical proliferative epithelial lesions	196	18.75
Adenomyoepitheliomas	13	1.25
Fibroepithelial lesions	220	21.05
- Fibroadenoma with stromal hypercellularity	176	16.84 (80.0)
- Fibroadenoma with epithelial atypia	16	1.53 (7.27)
- Phyllodes tumor	27	2.58 (12.25)
- Not specified	1	0.1 (0.45)
Stromal lesions	10	0.95
- Hemangioma	3	0.285 (30.0)
- Mesenchymal lesion/Fibromatosis	5	0.475 (50.0)
- Pseudoangiomatous stromal hyperplasia	2	0.19 (20.0)

**Table 4:** Histological features of epithelial proliferative B3 lesions.

	Nr. Lesions	Percentage (%)
Nodular proliferative epithelial lesions (without atypia)	537	51.4
- Intraductal papilloma	251	24 (46.7)
- Radial scar/complex sclerosing lesion	286	27.4 (53.3)
Nodular proliferative epithelial lesions (with atypia)	69	6.6
- Intraductal papilloma	26	2.5 (37.6)
- Radial scar/complex sclerosing lesion	43	4.1 (62.4)
Diffuse/non-nodular atypical proliferative epithelial lesions	196	18.75
- Atypical ductal hyperplasia	92	8.8 (47)
- Flat epithelial atypia	16	1.53 (8.2)
- Atypical lobular hyperplasia	65	6.22 (33.2)
- Lobular carcinoma in situ, classic type	21	2 (10.6)
- Atypical apocrine adenosis	1	0.1 (0.5)
- Atypical microglandular adenosis	1	0.1 (0.5)

**Table 5:** Upgrade Rates with 95% confidence intervals of epithelial proliferative B3 lesions.

B3 Lesions		Resection	Diagnostic Concordance		Diagnosis of Malignancy on Resection		Upgrade	
Type	Subtype		Nr.	Percentage (%)	Invasive Carcinoma	Ductal Carcinoma In Situ	Number Upgraded	Upgrade Rate (95% CI)
Nodular proliferative epithelial lesions (without atypia)	Intraductal PL	181	143	79.01	0	12 *	12	6.63 (3.5–11.3)
	RS-CSL	222	132	59.46	5	6	11	4.95 (2.5–8.7)
Subtotal		403	275	68.24	5	18	23	5.71 (3.7–8.4)
Nodular proliferative epithelial lesions (with atypia)	Intraductal PL	17	7	41.18	1	9 **	10	58.82 (32.9–81.6)
	RS-CSL	33	19	57.58	2	6 ***	8	24.24 (11.1–42.3)
Subtotal		50	26	52.0	3	15	18	36.0 (22.9–50.8)
Diffuse/non-nodular atypical proliferative epithelial lesions	ADH	65	17	26.15	5	20	25	38.46 (26.7–51.4)
	FEA	11	3	27.27	1	3	4	36.36 (10.9–69.2)
	ALH	46	32	69.57	5	3 ***	8	17.39 (7.8–31.4)
	LCIS	12	10	83.33	2	0	2	16.67 (2.1–48.4)
	AAA	1	1	100				
	AMGA	1	0		1		1	100
Subtotal		136	63	46.32	14	26	40	29.41 (21.9–37.8)

Note: * 8 papillary ductal carcinomas in situ, ** 5 papillary ductal carcinomas in situ, *** 1 florid lobular carcinoma in situ. Abbreviation: CI, confidence interval; PL, papilloma; RS-CSL, radial scar/complex sclerosing lesion; ADH, atypical ductal hyperplasia; FEA, flat epithelial atypia; ALH, atypical lobular hyperplasia; LCIS, lobular carcinoma in situ; AAA, Atypical apocrine adenosis; AMGA, Atypical microglandular adenosis.

**Table 6:** Upgrade Rates with 95% confidence intervals of remaining B3 lesions.

B3 Lesions		Resection	Diagnostic Concordance		Outcome *		Upgrade	
Type	Subtype		Nr.	Percentage (%)	Borderline PT	Malignant PT	Number Upgraded	Upgrade Rate (95% CI)
Fibroepithelial lesions	FA with epithelial atypia	11	9	81.82	0		0	
	FA with hypercellular stroma	148	122	82.43	2		2	1.35 (0.24–4.79)
	PT	25	19	76.0	4	2	6	24.0 (9.4–45.1)
	Not specified	1	0		0	0		
Subtotal		185	150	81.08	6	2	8	4.32 (2.21–8.3)
Stromal lesions	Hemangioma	2	2	100				
	Mesenchymal lesion/Fibromatosis	4	4	100				
	PASH	2	1	50				
Subtotal		8	7	87.5				
Other lesions	Adenomyoepitheliomas	13	8	61.54				

Note: * no upgrade included an epithelial lesion (example, invasive carcinomas or ductal carcinoma in situ). Abbreviation: PT, phyllodes tumor; CI, confidence interval; FA, fibroadenoma; PASH, pseudoangiomatous stromal hyperplasia.

**Table 7:** Significant results between the upgrade rates of diffuse/non-nodular atypical proliferative B3 epithelial lesions.

	ADH	FEA	ALH	LCIS	AMGA
ADH					
FEA	No				
ALH	Yes, *p* = 0.0206	No			
LCIS	No	No	No		
AMGA	No	No	No	No	

Abbreviation: ADH, atypical ductal hyperplasia; FEA, flat epithelial atypia; ALH, atypical lobular hyperplasia; LCIS, lobular carcinoma in situ; AMGA, atypical microglandular adenosis.

**Table 8:** Histological features of upgrade invasive carcinomas.

Type	Nr.	Grading			Luminal A	Luminal B	HER2	TNBC
		G1	G2	G3				
Invasive breast carcinomas of no special type	12	7	5		11	1	0	0
Invasive lobular carcinomas	4		3 *		4	0	0	0
Mixed invasive ductal lobular carcinomas	2		2		2			
Tubular carcinomas	1	1			1			
Carcinomas with apocrine differentiation	1		1					1
Mucinous carcinomas	1		1		1			
Metaplastic carcinomas	1			1				1
Total	22	8	12	1	19	1		2

Note: * 1 data not available. All invasive carcinomas were ipsilateral. Abbreviation: G, grade; HER2, human epidermal growth factor receptor 2; TNBC, triple-negative breast cancer.

## Data Availability

Dataset available on request from the authors.
